# Circulating microRNAs related to cognitive performance in the general population

**DOI:** 10.1002/alz.083648

**Published:** 2025-01-03

**Authors:** Konstantinos Melas, Valentina Talevi, Dennis Manfred Krüger, Tonatiuh Pena, N. Ahmad Aziz, André Fischer, Monique M.B. Breteler

**Affiliations:** ^1^ German Center for Neurodegenerative Diseases (DZNE), Bonn, North Rhine‐Westphalia Germany; ^2^ German Centre for Neurodegenerative Diseases (DZNE), Göttingen, Lower Saxony Germany; ^3^ Faculty of Medicine, University of Bonn, Bonn, North Rhine‐Westphalia Germany; ^4^ Population Health Sciences, German Center for Neurodegenerative Diseases (DZNE), Bonn Germany; ^5^ University Medical Center Göttingen, Göttingen, Lower Saxony Germany; ^6^ Cluster of Excellence MBExC, Göttingen, Lower Saxony Germany; ^7^ Institute for Medical Biometry, Informatics and Epidemiology (IMBIE), Faculty of Medicine, University of Bonn, Bonn Germany

## Abstract

**Background:**

MicroRNAs have been linked to dementia. However, understanding their relation to cognition in the general population is required to determine their potential use for the detection and prevention of age‐associated cognitive decline and preclinical dementia. Therefore, we examined the association of circulating microRNAs with cognitive performance in a population‐based cohort and the possible underlying mechanisms.

**Method:**

We used data from 2869 participants (55.6% women, mean age: 55.01 years, age range: 30 to 95 years) of the Rhineland Study, a prospective population‐based study in Bonn, Germany. Circulating microRNA and gene expression were measured through RNA‐Seq and microRNAs were clustered using weighted gene co‐expression network analysis. Cognitive domain scores were derived from a neuropsychological test battery. Brain imaging measures were obtained using Freesurfer on 3T T1‐weighted MR images. We employed multivariable linear regression to evaluate the association of cluster and individual microRNA expression levels with cognitive domain scores, imaging measures, and target gene expression, adjusting for multiple testing with the false discovery rate (fdr) method.

**Result:**

One microRNA cluster, represented by miR‐134‐5p, miR‐409‐3p, miR‐370‐3p, and miR‐493‐3p, was significantly associated with executive function (standardized β: ‐0.045, fdr‐adjusted p‐value: 0.043). Another cluster, represented by hub microRNAs miR‐215‐5p and miR‐192‐5p, was associated with episodic verbal memory at suggestive significance (standardized β: ‐0.040, fdr‐adjusted p‐value: 0.097). When examining individual microRNAs, miR‐92b‐3p, miR‐1976, miR‐4677‐5p, and miR‐10401‐3p were significantly associated with executive function and global cognition. Importantly, miR‐215‐5p, miR‐192‐5p, miR‐92b‐3p, miR‐1976, and miR‐10401‐3p were also associated with cortical thickness, predominantly in the temporal and frontal regions. Functional enrichment analysis of microRNA target genes uncovered involvement of pathways related to brain and dendrite development, axon guidance and synapse assembly.

**Conclusion:**

We identified microRNA clusters and individual microRNAs that were related to different cognitive functions. Some of these microRNAs were also related to cortical thickness, suggesting that part of their relation with cognition is mediated through alterations of brain structure. The identified microRNAs regulated genes related to brain homeostasis and development. Our results contribute to a better understanding of brain epigenetics and provide potential molecular targets for the detection, prevention or treatment of cognitive dysfunction.

